# Melatonin Restores the Developmental Competence of Heat Stressed Porcine Oocytes, and Alters the Expression of Genes Related to Oocyte Maturation

**DOI:** 10.3390/ani11041086

**Published:** 2021-04-10

**Authors:** Ling Yang, Zimo Zhao, Maosheng Cui, Leying Zhang, Qianjun Li

**Affiliations:** 1 School of Life Sciences and Food Engineering, Hebei University of Engineering, Handan 056038, China; yangling@hebeu.edu.cn (L.Y.); zhaozm0320@126.com (Z.Z.); zhangly056000@126.com (L.Z.); 2 Animal Husbandry and Veterinary Research Institute of Tianjin, Tianjin 300412, China; tjlqj@sina.com

**Keywords:** melatonin, in vitro maturation, oocyte, pig

## Abstract

**Simple Summary:**

Melatonin improves the quality and in vitro maturation (IVM) of oocytes under heat stress. Melatonin treatment counteracts the adverse effects induced by heat stress (HS), such as the poor survival rate and maturation rate, distribution of α-tubulin and F-actin, expression of *NRF2* and *GDF9* mRNA. However, HS and melatonin have similar effects on increasing expression of *HSP70* and *NRF2* mRNA. Furthermore, HS inhibits expression of *GDF9* mRNA.

**Abstract:**

Melatonin enhances the quality and in vitro maturation (IVM) of oocytes under heat stress (HS), but the mechanism of melatonin in reducing HS injury on oocytes is not fully understood. In this study, porcine cumulus-oocyte complexes (COCs) were randomly divided into three groups. The COCs of the control group were cultured at 38.5 °C for 42 h, and the COCs of the HS group were cultured at 41.5 °C for 4 h, and then transferred into 38.5 °C for 38 h. The COCs of the HS + melatonin group were cultured with 10^−9^ M melatonin under the same conditions as the HS group. The survival rate, maturation rate, distribution of α-tubulin and F-actin of the oocytes were assessed. In addition, the expression profiles for genes related to the oocyte maturation, including heat shock protein 70 (*HSP70*), nuclear factor erythroid 2-related factor 2 (*NRF2*), cyclin-dependent kinase 1 (*CDK1*), growth differentiation factor 9 (*GDF9*) were analyzed by real-time quantitative PCR. The results showed that HS decreased the survival rate and maturation rate, distribution of α-tubulin and F-actin, but melatonin treatment could partly counteract these adverse effects. In addition, HS increased expression of *HSP70* and *NRF2* mRNA, and melatonin treatment had a similar effect on *HSP70* expression, but had a contrary effect on *NRF2* expression. Furthermore, HS inhibited expression of *CDK1* and *GDF9* mRNA, but melatonin treatment could weaken the effect on *GDF9* expression induced by HS. In summary, melatonin treatment could attenuate the unfavorable effects induced by HS to enhance developmental competence of porcine oocytes during IVM.

## 1. Introduction

There is a decrease in fertility of dairy cows during the summer, and the adverse effects of summer heat stress (HS) on oocyte quality can be improved via removal of the impaired cohort of follicles in the autumn [1]. Sows during cooler months have shorter weaning-to-service intervals, higher farrowing rates and larger born and weaned litter sizes than the sows during high ambient temperature, which indicates that high ambient temperature has unfavorable effects on porcine reproductive performance [2]. The fertility of female rabbits decreases, but a quercetin-supplemented diet reduces ovarian apoptosis, and maintains the oocyte competence during summer HS [3]. HS has deleterious effects on the developmental potential of oocytes, including oocyte cytoplasmic maturation and meiotic competence, in pigs and mice [4,5]. In vitro maturation (IVM) is used routinely in the in vitro production of embryos for the research and production of offspring in breeding programs in domestic species [6]. HS induces ceramide generation to cause reactive oxygen species (ROS) production and mitochondrial dysfunction, which reduce the proportion of cumulus-oocyte complexes (COCs) exhibiting fully expanded cumulus cells and the rate of metaphase II in porcine oocytes [7]. HS has significant effects on the nucleus and cytoskeleton of IVM bovine oocytes, which is related to the low pregnancy rates in domestic species during hot seasons [8].

High-quality IVM oocytes maintain a round shape, and mis-shapened oocytes have an abnormal organization of cytoskeletal components and low developmental competence in pigs [9]. The cytoskeleton, including microfilaments and microtubules, is involved in determining the shape and intra-cellular movement of organelles in mammalian oocytes, and plays key roles in regulating porcine oocyte maturation [10]. HS obviously reduces oocyte quality, and causes a decrease in the density of filamentous actin (F-actin) in the zona pellucida during oocyte maturation in pigs [11]. Growing oocytes can synthesize heat shock protein 70 (HSP70) after heat shock, and HSP70 plays key roles in oocyte growth and maturation in pigs [12]. Nuclear factor erythroid 2-related factor 2 (Nrf2) is a key transcription factor, and is implicated in oocyte maturation and spindle/chromosome organization in oocyte meiosis through the Nrf2-Cyclin B1 pathway in mice [13]. Cyclin-dependent kinase 1 (CDK1) acts as the kinase subunit of a maturation-promoting factor, and is related to cumulus cell expansion and germinal vesicle breakdown during porcine oocyte maturation [14]. Growth differentiation factor 9 (GDF9) is a member of the transforming growth factor-beta superfamily, and oocytes can secrete GDF9 to regulate its quality and developmental competence in humans [15]. There is an upregulation of GDF9 in the porcine oocytes after progesterone treatment, which is beneficial for improving porcine COC development and subsequent embryo development through the epidermal growth factor receptor signaling pathway [16].

Melatonin not only works as a circadian rhythm modulator, but also acts as a direct free radical scavenger, indirect antioxidant and cytoprotective agent during pregnancy in humans [17]. Our recent study showed that melatonin treatment enhances quality of porcine IVM oocytes though antioxidant and anti-apoptotic mechanisms [18]. It has been reported that melatonin application reduces ROS formation, improves glutathione (GSH) production, suppresses cell apoptosis, and enhances expression of Sirtuin 1, AKT serine/threonine kinase 2 and DNA polymerase gamma 2 genes, to protect the IVM of porcine oocytes under HS [19]. Melatonin treatment reduces the production of ROS in the maturing oocyte, and protects oocytes from deleterious effects of HS [20]. However, the exact mechanisms through which melatonin protects the IVM oocyte from HS have not been fully understood. In this study, the effects of melatonin on oocyte survival rate, maturation rate, formation of F-actin and α-tubulin, and expression of *HSP70*, *NRF2*, *CDK1* and *GDF9* genes were assessed after HS in pigs. The results will provide valuable information for using melatonin to enhance the oocyte IVM under HS in humans and animals.

## 2. Materials and Methods

### 2.1. Animal Studies

All animal studies were in accordance with the requirement of the Institutional Animal Care Committee, and approved (AHVRIT-2015049) and conducted under the requirements of the Animal Husbandry and Veterinary Research Institute of Tianjin, China.

### 2.2. Oocytes Collection and In Vitro Maturation

Porcine ovaries were collected from a local abattoir (Xiqing District, Tianjin, China) and shipped immediately to the laboratory within 2 h in a thermos (35–37 °C). COCs were aspirated from the follicles (2–8 mm in diameter), and the oocytes were selected for IVM with a homogeneous cytoplasm and at least three intact layers of surrounding cumulus cells. Porcine follicle fluid (pFF) was aspirated from the follicles, centrifuged at 2000× *g* for 10 min, and the supernatant was filtered through a 0.22 μm membrane (MILLEX^®^-HV; Millipore, Billerica, MA, USA). COCs were washed three times in a pre-warmed IVM medium that consisted of M199 medium (Gibco, Life Technology, Carlsbad, CA, USA) with follicle-stimulating hormone (0.01 IU/mL) and luteinizing hormone (0.01 IU/mL), 1 IU/mL antibiotics (penicillin/streptomycin; Gibco) and 20% pFF, and randomly divided into three groups (control group, HS group, and HS + melatonin group). The COCs of the control group were cultured in 100 μL of the maturation medium under mineral oil at 38.5 °C, 5% CO_2_ in air with 100% humidity for 42 h. The COCs of the HS group were cultured in the same maturation medium at 41.5 °C [11] for 4 h, and then transferred into 38.5 °C for 38 h. The COCs of the HS + melatonin (HSMT) group were cultured in the maturation medium supplemented with 10^−9^ M melatonin [21] under the same conditions as the HS group.

### 2.3. Assessment of Oocyte Survival Rate and Maturation Rate

The surviving oocyte is the oocyte with intact zona pellucida and plasma membrane, and the space between zona pellucida and cell membrane was clear without cytoplasmic leakage or oocyte shrinkage. Oocyte survival rate is defined as the ratio of the number of surviving oocytes to the number of oocytes that underwent IVM. The oocytes were stained with Hoechst 33342 after the cumulus cells were removed as described previously [22]. The stained oocytes were examined with an inverted microscope (Nikon Corporation., Tokyo, Japan) equipped with epifluorescence, and mature oocytes were determined by the presence of the first polar body. The maturation rate was the number of mature oocytes/total number of the oocytes × 100%.

### 2.4. Immunofluorescence, α-Tubulin and F-Actin Staining

Oocytes used for examination of F-actin and α-tubulin were fixed by 4% paraformaldehyde in PBS for 45 min at room temperature. The oocytes (*n* = 30 for each group) were then transferred to 1% Triton X-100 in PBS for 6 h at room temperature. After being washed thrice (10 min each) in PBS, the oocytes were blocked with 1% bovine serum albumin in PBS at 4 °C overnight. For α-tubulin staining, the oocytes were subsequently incubated with a rabbit anti-α-tubulin antibody (11H10, #2125S, Cell Signaling Technology, Danvers, USA, 1:50) at 4 °C overnight. After being washed thrice (10 min each), the oocytes were incubated with a goat anti-rabbit IgG H and L antibody (Alexa Fluor^®^ 594, Abcam, Cambridge, UK, ab150080) for 1 h at room temperature. The nuclear status of the oocyte was checked using staining fluorescent dye Hoechst 33342 (C1022, Beyotime, Jiangsu, China) according to the manufacturer’s instructions. For F-actin staining, after being washed thrice (10 min each), the samples were stained using Alexa Fluor 488 phalloidin (A12379, Thermo Fisher Scientific, Wilmington, MA, USA) at a final concentration of 100 ng/mL. Finally, after three washes in washing buffer, the oocytes were mounted on glass slides, and examined using a confocal laser-scanning microscope (Leica Microsystems, Heidelberg, Germany).

### 2.5. RNA Extraction and Real-Time Quantitative PCR (RT-qPCR)

The cumulus-stripped oocytes from the control, HS and HSMT groups (*n* = 100 for each group) were lysed using Trizol reagent (Invitrogen, California, CA, USA), and total RNA was isolated according to the manufacturer’s instructions. DNase-I (GeneCopoeia, Rockville, MD, USA) was used to remove genomic DNA, and a first-strand cDNA synthesis kit (GeneCopoeia) was used to synthesize cDNA following the manufacturer’s instructions. The primer sequences (Table 1) of *HSP70*, *NRF2*, *CDK1*, *GDF9* and *GAPDH* genes were designed and synthesized by Shanghai Sangon Biotech Co., Ltd. (Shanghai, China). Amplification efficiencies of the primer sequences were evaluated before quantification, and were in an acceptable range (between 0.9 and 1.1). A 7900HT System (Applied Biosystems, Foster City, CA, USA) was used for qPCR with a qPCR detection kit (GeneCopoeia). The expression of *GAPDH* was used for normalization, and the relative expression levels of *HSP70*, *NRF2*, *CDK1* and *GDF9* mRNA were determined by the 2^−ΔΔCt^ analysis method [23]. GAPDH has been found to be stable in porcine oocyte-granulosa cell complexes, and can be used as a housekeeping gene [24].

### 2.6. Statistical Analysis

The fluorescence intensity was determined using ImageJ software by the National Institutes of Health (NIH, Bethesda, MD, USA) as described previously [25]. Each group consisted of three biological replicates, and the data were presented as the mean ± SEM. Data normality and homoscedasticity were tested using the PROC UNIVARIATE procedure in SAS version 8 (SAS Institute Inc., Cary, NC, USA). One-way ANOVA followed by Duncan’s test was used for equal variances while Kruskal–Wallis one-factor ANOVA was performed to compare means with unequal variances. *p* < 0.05 indicated a significant difference.

## 3. Results

### 3.1. Effects of HS and Melatonin on Oocyte Survival Rate and Maturation Rate

The data showed that the oocyte survival rate of the control group was higher than that of the HS group and HSMT group (*p* < 0.05; Table 2), but there was no significant difference between the HS group and HSMT group (*p* > 0.05; Table 2). In addition, the maturation rates of the control group and HSMT group were significantly higher than that of the HS group (*p* < 0.05; Table 2). HS induced a significant decrease in the survival rate and maturation rate of the oocyte, but melatonin could efficiently improve the maturation rate of HS-treated oocytes.

### 3.2. Effect of HS and Melatonin on the α-Tubulin Distribution in the Porcine Oocytes

**Figure 1:**
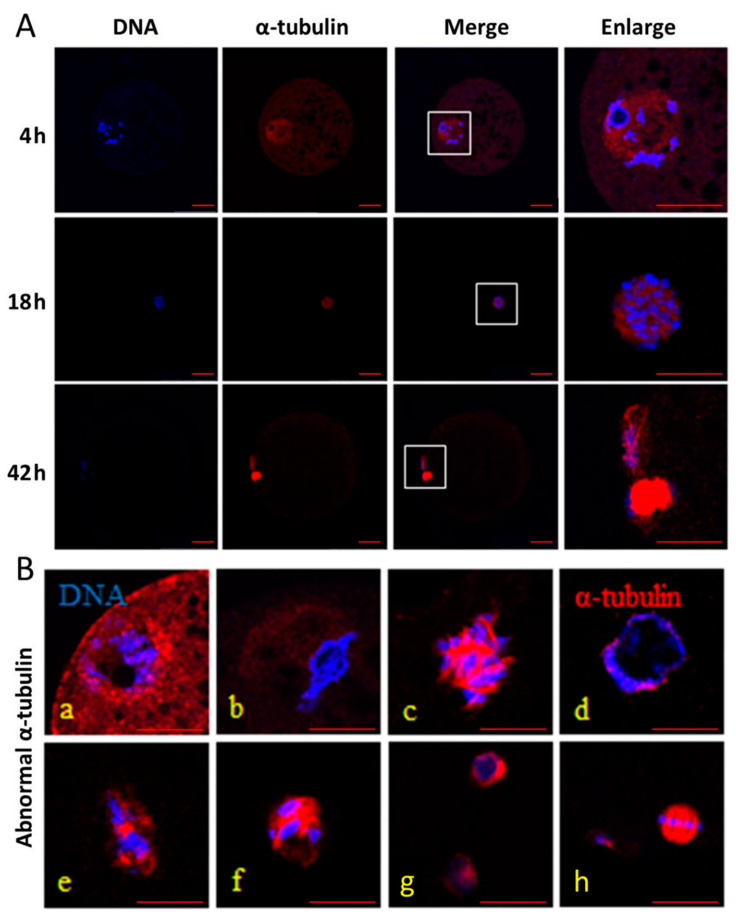
Effect of heat stress (HS) and melatonin on the normal rate of α-tubulin in the porcine oocytes during in vitro maturation (IVM). DNA (blue) is stained by Hoechst 33342, and α-tubulin (red) is stained using an anti-α-tubulin antibody. (**A**): normal α-tubulin and DNA in the oocytes during IVM at 4, 18 and 42 h, respectively. (**B**): abnormal α-tubulin and DNA in the oocytes during IVM (**a**,**b** for 4 h; **c**–**f** for 18 h; **g** and **h** for 42 h). Bar = 20 μm.

It was shown in Figure 1 that α-tubulin was almost evenly distributed in the cytoplasm during IVM at 4 h, and began to accumulate in the vicinity of the condensed chromosomes during IVM at 18 h. Finally, α-tubulin formed spindles and enriched at the equatorial plate during IVM at 42 h. In addition, there was no significant difference in the normal rates of α-tubulin among the three groups during IVM at 4 h (*p* > 0.05; Table 3), but the normal rate in the control group was the highest among the three groups during IVM at 18 and 42 h (*p* < 0.05). The normal rate was the lowest in the HS group among the three groups (*p* < 0.05), and melatonin treatment could attenuate the unfavorable effects on the normal rate induced by HS during IVM at 18 and 42 h.

### 3.3. Effect of HS and Melatonin on F-Actin Distribution in the Porcine Oocytes

**Figure 2:**
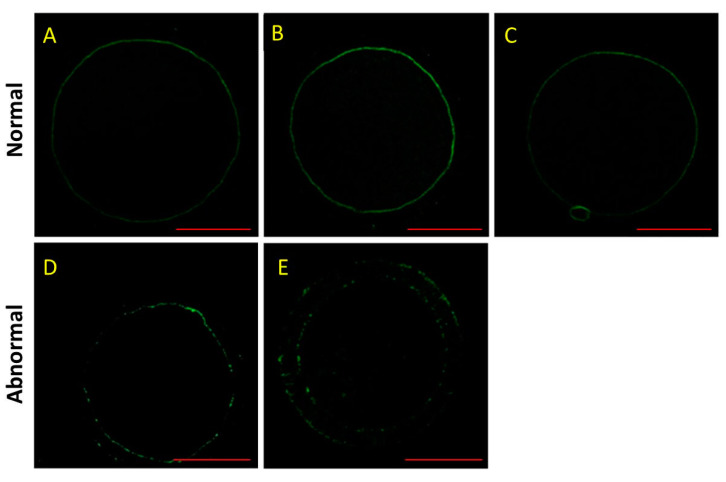
Effect of heat stress (HS) and melatonin on the normal rate of F-actin in the porcine oocytes during in vitro maturation (IVM). F-actin is stained using phalloidin (green). (**A**–**C**) are the representative images of normal F-actin in the porcine oocytes during IVM at 4, 18 and 42 h, respectively. (**D,E**) are the representative images of abnormal F-actin in porcine oocytes during IVM. Bar = 50.

As shown in Figure 2, the normal F-actin was in a continuous distribution, but the abnormal F-actin had a decrease in fluorescence intensity with a discontinuous distribution. In addition, the F-actin was located in transzonal projections that constituted a structural basis for the communication between the oocyte and its surrounding cumulus cells as described previously [26]. Furthermore, there were significant decreases in the normal rates of F-actin in the HS group and HSMT group during IVM (*p* < 0.05; Table 4). Melatonin treatment could improve the normal rate of F-actin after HS during IVM at 18 and 42 h (*p* < 0.05).

### 3.4. Effect of HS and Melatonin on Expression of HSP70, NRF2, CDK1 and GDF9 mRNA in the Porcine Oocytes during IVM

**Figure 3:**
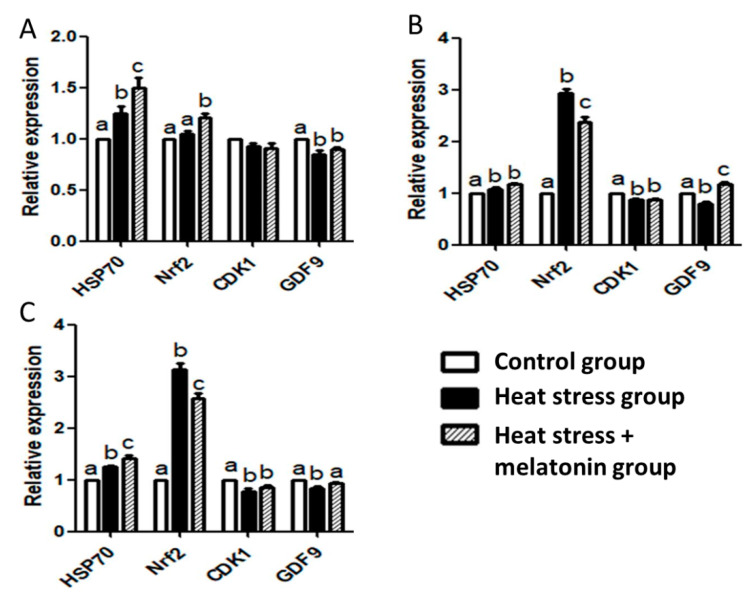
Effect of heat stress (HS) and melatonin on expression of *HSP70*, *NRF2*, *CDK1* and *GDF9* mRNA in the porcine oocytes during in vitro maturation (IVM). (**A**): effect of HS and melatonin on the oocytes during IVM at 4 h; (**B**): effect of HS and melatonin on the oocytes during IVM at 18 h; (**C**): effect of HS and melatonin on the oocytes during IVM at 42 h. Different letters: ^a,b,c^ in the different patterned columns represent significant difference (*p* < 0.05).

The RT-qPCR results showed (Figure 3) the HS-induced upregulation of *HSP70* mRNA in the oocytes during IVM (*p* < 0.05). HS and melatonin had cooperative effects on improving the expression of *HSP70* mRNA during IVM at 4 and 42 h (*p* < 0.05). HS increased the expression of *NRF2* mRNA (*p* < 0.05), but melatonin suppressed the effects of HS on the expression of *NRF2* mRNA (*p* < 0.05) during IVM at 18 and 42 h. HS downregulated expression of *CDK1* mRNA in the oocytes during IVM from 18 to 42 h (*p* < 0.05), but melatonin had no significant effect on the expression of *CDK1* mRNA (*p* > 0.05). HS caused a decrease in the expression of *GDF9* mRNA in the oocytes during IVM (*p* < 0.05), but melatonin could efficiently inhibit the effects of HS during IVM from 18 to 42 h (*p* < 0.05). Furthermore, melatonin supplements could enhance the expression of *GDF9* mRNA during IVM at 18 h (*p* < 0.05).

## 4. Discussion

Our results indicated that HS had adverse effects on the survival rate and maturation rate of the oocytes during IVM, but melatonin could counteract the unfavorable effect on the maturation rate of the oocyte. As an antioxidant and anti-apoptotic agent, melatonin enhances in vitro gamete and embryo development in mammals [27]. Melatonin improves the developmental competence of the oocyte–granulosa cell complexes through modulating metabolic pathways in pigs [24]. Our previous study indicates that melatonin treatment enhances the quality and in vitro development of porcine oocytes via the antioxidant and anti-apoptotic mechanisms [18]. It has been reported that melatonin reduces ROS formation, enhances GSH production and polar body rate of the porcine oocytes under the HS during IVM [19]. Therefore, it is confirmed in this study that melatonin supplementation is beneficial for oocyte IVM under HS.

It was shown in this study that melatonin treatment had favorable effects on the α-tubulin distribution in the porcine oocytes after HS. Meiotic drive in oocytes is related to microtubule force asymmetry on chromosomes, including inherent asymmetry in tubulin density, and tubulin asymmetry plays key roles in the spindle migration [28]. Transcription factor IIB is a target of various transcriptional activator proteins, and interacts with α-tubulin during oocyte meiosis, which participates in subsequent embryo development in mice [29]. Tubulin post-translational modifications are related to the spindle organization and IVM of prepubertal and adult oocytes in sheep [30]. There are changes in α-tubulin distribution during mitosis and meiosis, such as polymerization in the cell cortex and becoming two polarized arrays in pig oocytes [31]. Fenoxaprop-ethyl exposure results in disruption of cytoskeletal integrality, and melatonin can lessen these meiotic defects and has favorable effects on oocytes during meiotic maturation in mice [32]. HS treatment induces abnormalities in the chromosomes and spindle tubulin, which reduces development of the porcine oocytes [33]. In this study, melatonin treatment improves oocyte maturation rate, which may be partly through improving the normal rate of α-tubulin in the porcine oocytes after HS.

Our data indicated that HS caused significant decreases in the normal rate of F-actin during IVM, but melatonin treatment had beneficial effects on the F-actin distribution in the porcine oocytes after HS. F-actin is rich in the oocyte cortex, which is involved in sperm entry and transient harboring during fertilization [26]. Tyrosine kinase 2 recruitment and activation in the oocyte cortex induces F-actin accumulation and remodeling, which is necessary for sperm incorporation and sperm going into the ooplasm [34]. F-actin is implicated in the intercellular communication between cumulus cells and the oocyte during oocyte maturation, and HS disrupts F-actin and gap junction protein connexin-45 colocalization in porcine cumulus cells and oocytes [35]. HS induces a decrease in the fluorescence intensity of F-actin in the zona pellucida during oocyte maturation, which has adverse effects on the developmental competence of oocytes in pigs [11]. The actin cap is disrupted in the subcortex of aged oocytes, and melatonin restores formation of the actin cap to improve the fertilization ability of post-ovulatory aged oocytes in mice [36]. Therefore, melatonin may improve developmental competence of oocytes partly through restoring the F-actin distribution in the porcine oocytes after HS.

Our results revealed that HS induced *HSP70* expression, and melatonin also improved *HSP70* expression in the oocytes during IVM at 4 and 42 h. HSP70 functions as a molecular chaperone, and can prevent DNA strand breaks, protecting mitochondrial structure [37]. There are great amounts of HSP70 proteins in fully grown oocytes without heat shock treatment, and growing oocytes can synthesize HSP70 after heat shock in pigs [12]. HSP70 is important for oocyte growth and maturation. Oocytes are incubated in IVM media with coagulansin-A, a steroidal lactone, which increases HSP70 expression and thermotolerance during in vitro production of bovine embryos [38]. A natural higher production of HSP70 protein in the oocyte participates in the mechanisms of adaptation to heat conditions in Pantaneira cattle [39]. *HSP70* mRNA increases two- to threefold between the one- and two-cell stages of embryos, which is essential for the zygotic gene activation in mice [40]. There is an increase in expression of *HSP70* mRNA in the embryos derived from melatonin-treated oocytes, and HSP70 plays important roles in oogenesis and genesis in cattle [41]. There is a decline in developmental competence of porcine-matured oocytes after HS, however, which is not closely associated with the expression of *HSP70* [42]. Therefore, the upregulation of *HSP70* induced by HS and melatonin may be not closely associated with the decrease in developmental competence of the pig oocytes, and further research may be needed.

It was found in this study that HS stimulated the expression of *NRF2* mRNA, but melatonin attenuated the expression of *NRF2* mRNA induced by HS. Nrf2 has antioxidant effects through scavenging excessive ROS and restoring redox homeostasis, but uncontrolled Nrf2 activation can lead to harmful consequences [43]. Nrf2 depletion has negative effects on oocyte maturation and spindle/chromosome organization, and the decline in the reproductive capacity of older women is related to the downregulation of Nrf2 [44]. Expression of *NRF2* mRNA peaks at 24 h post-HS in liver, which causes hepatocyte abnormalities [45]. HS has negative effects on bovine reproduction, and induces oxidative stress, and enhances Nrf2 expression in bovine granulosa cells [46]. Melatonin treatment induces upregulation of Nrf2 protein, which is involved in antioxidative stress response in the porcine in vitro fertilization embryo [47]. Brusatol (an Nrf2 specific inhibitor) inhibits expression of Nrf2, and modulates Cyclin B1 level to disturb proper spindle assembly and chromosome condensation in mouse oocytes [13]. Melatonin increases the Nrf2 expression, but Brusatol neutralizes the effects on Nrf2 expression induced by melatonin in porcine COCs [48]. Therefore, HS may induce overexpression of *NRF2*, and have negative effects on the porcine oocytes, but melatonin treatment may have beneficial effects through attenuating *NRF2* overexpression.

This study indicated that HS inhibited *CDK1* expression in the oocytes during IVM from 18 to 42 h, but melatonin had no significant effect on *CDK1* expression. CDK1 activation is related to cyclin B2 degradation, which is essential for chromosome segregation during the oocyte meiosis G2/M transition in mice [49]. CDK1 is sensitive to HS in human neuroblastoma and glioma cell lines, and related to protein homeostasis in mammalian cells [50]. Melatonin can inhibit human osteosarcoma cell proliferation through downregulation of CDK1 [51]. Therefore, melatonin may have beneficial effects, but have no significant effect on the expression *CDK1* induced by HS.

This study demonstrated that HS inhibited expression of *GDF9* mRNA in the oocytes, but melatonin could neutralize this effect induced by HS during IVM. GDF9 secreted by the oocyte is involved in regulating the function of granulosa and thecal cells, which is related to the oocyte quality in humans [52]. Serum GDF9 concentration is associated with female reproductive potential in humans [53]. Co-culture canine oviduct cells with porcine oocytes induce upregulation of *GDF9* mRNA, which is beneficial for oocytes during IVM [54]. The relative abundance of *GDF9* mRNA is lower in the bovine oocytes collected in the hot season than that in the cold season [55]. Melatonin treatment improves *GDF9* mRNA expression via melatonin membrane receptors of oocytes, which is helpful for bovine oocytes during IVM [56]. Therefore, HS suppressed *GDF9* expression, and had adverse effects on oocytes during IVM. Melatonin had positive effects partly through enhancing *GDF9* expression in pigs.

## 5. Conclusions

HS had adverse effects on the survival rate and maturation rate, distribution of α-tubulin and F-actin of the oocytes, but melatonin treatment could partly counteract these adverse effects during IVM. In addition, HS induced upregulation of *HSP70* and *NRF2* mRNA. Melatonin treatment had a similar effect on *HSP70* expression, but had a contrary effect on *NRF2* expression in the oocytes during IVM. Furthermore, HS inhibited expression of *CDK1* and *GDF9* mRNA. Melatonin treatment had no significant effect on *CDK1* expression, but could neutralize the effect on *GDF9* expression induced by HS in the porcine oocytes during IVM. In summary, HS had adverse effects on oocyte survival rate, maturation rate, formation of F-actin and α-tubulin, and expression of *NRF2*, *CDK1* and *GDF9* genes, but melatonin treatment could attenuate these unfavorable effects to improve developmental competence of oocytes during IVM in pigs.

## Figures and Tables

**Table 1:** Primer sequences for RT-qPCR analysis.

Gene	Primer Sequence (5′–3′)	Product Size (Bp)	GenBank Accession No.
*GAPDH*	F: TCAAATGGGGTGATGCTGGT R: GCAGAAGGGGCAGAGATGAT	124	XM_021091114
*GDF-9*	F: CCTCTACAACACTGTCCGGC R: GTCCCCTGATGGAAGGGTTC	91	NM_001001909.1
*CDK1*	F: TAATAAGCTGGGATCTACCACATC R: TGGCTACCACTTGACCTGTA	130	NM_001159304
*HSP70*	F: TGAATCCGCAGAATACCGTG R: CTCCGCAGTCTCCTTCATC	215	NM_001123127.1
*NRF2*	F: AAGCCTTCAACCAAGACCA R: AGAATCACTGAAGCCAAGCA	179	MH101365.1

**Table 2:** Effects of heat stress and melatonin on oocyte survival rate and maturation rate.

Group	Number of Oocytes	Replicates	Survival Rate (%)	Maturation Rate (%)
Control	276	3	96.86 ^b^ ± 2.14	72.4 ^b^ ± 2.13
HS	252	3	88.72 ^a^ ± 3.42	59.6 ^a^ ± 3.06
HSMT	282	3	90.23 ^a^ ± 2.85	68.3 ^b^ ± 1.95

Note: HS: heat stress; HSMT: heat stress + melatonin. Significant differences (*p* < 0.05) are indicated by different letters within the same column: ^a,b^.

**Table 3:** Normal rate of α-tubulin (%).

Group	4 h	18 h	42 h
Control	75.56 ± 4.84	76.67 ^a^ ± 3.85	77.78 ^a^ ± 4.45
HS	73.33 ± 1.93	41.11 ^b^ ± 2.94	43.33 ^b^ ± 3.85
HSMT	72.22 ± 3.68	62.22 ^c^ ± 2.94	61.11 ^c^ ± 4.01

HS: heat stress; HSMT: heat stress + melatonin. Significant differences (*p* < 0.05) are indicated by different letters within the same column: ^a,b,c^.

**Table 4:** Normal rate of F-actin (%).

Group	4 h	18 h	42 h
Control	84.44 ^a^ ± 1.11	83.33 ^a^ ± 1.87	85.56 ^a^ ± 2.94
HS	53.33 ^b^ ± 1.92	55.56 ^b^ ± 1.11	58.89 ^b^ ± 4.01
HSMT	54.45 ^b^ ± 4.01	66.67 ^c^ ± 3.85	71.11 ^c^ ± 2.22

HS: heat stress; HSMT: heat stress + melatonin. Significant differences (*p* < 0.05) are indicated by different letters within the same column: ^a,b,c^.

## Data Availability

The data presented in this study are available on request from the corresponding author. The data are not publicly available due to privacy or ethical restrictions.

## References

[1] Roth Z., Arav A., Bor A., Zeron Y., Braw-Tal R., Wolfenson D. (2001). Improvement of quality of oocytes collected in the autumn by enhanced removal of impaired follicles from previously heat-stressed cows. Reproduction.

[2] Boma M.H., Bilkei G. (2006). Seasonal infertility in Kenyan pig breeding units. Onderstepoort J. Vet. Res..

[3] Naseer Z., Ahmad E., Epikmen E.T., Uçan U., Boyacioğlu M., İpek E., Akosy M. (2017). Quercetin supplemented diet improves follicular development, oocyte quality, and reduces ovarian apoptosis in rabbits during summer heat stress. Theriogenology.

[4] Wang J.Z., Sui H.S., Miao D.Q., Liu N., Zhou P., Ge L., Tan J.H. (2009). Effects of heat stress during in vitro maturation on cytoplasmic versus nuclear components of mouse oocytes. Reproduction.

[5] Nishio K., Yamazaki M., Taniguchi M., Besshi K., Morita F., Kunihara T., Tanihara F., Takemoto T., Otoi T. (2017). Sensitivity of the meiotic stage to hyperthermia during in vitro maturation of porcine oocytes. Acta Vet. Hung..

[6] Lonergan P., Fair T. (2016). Maturation of oocytes in vitro. Annu. Rev. Anim. Biosci..

[7] Lee S., Kang H.G., Jeong P.S., Kim M.J., Park S.H., Song B.S., Sim B.W., Kim S.U. (2021). Heat stress impairs oocyte maturation through ceramide-mediated apoptosis in pigs. Sci. Total Environ..

[8] Roth Z., Hansen P.J. (2005). Disruption of nuclear maturation and rearrangement of cytoskeletal elements in bovine oocytes exposed to heat shock during maturation. Reproduction.

[9] Dang-Nguyen T.Q., Nguyen H.T., Somfai T., Wells D., Men N.T., Viet-Linh N., Noguchi J., Kaneko H., Kikuchi K., Nagai T. (2018). Sucrose assists selection of high-quality oocytes in pigs. Anim. Sci. J..

[10] Sun Q.Y., Lai L., Park K.W., Kühholzer B., Prather R.S., Schatten H. (2001). Dynamic events are differently mediated by microfilaments, microtubules, and mitogen-activated protein kinase during porcine oocyte maturation and fertilization in vitro. Biol. Reprod..

[11] Yin C., Liu J., Chang Z., He B., Yang Y., Zhao R. (2020). Heat exposure impairs porcine oocyte quality with suppressed actin expression in cumulus cells and disrupted F-actin formation in transzonal projections. J. Anim. Sci. Biotechnol..

[12] Lánská V., Chmelíková E., Sedmíková M., Petr J., Rajmon R., Jeseta M., Rozinek J. (2006). Expression of heat shock protein70 in pig oocytes: Heat shock response during oocyte growth. Anim. Reprod. Sci..

[13] Ma R., Li H., Zhang Y., Lin Y., Qiu X., Xie M., Yao B. (2017). The toxic effects and possible mechanisms of Brusatol on mouse oocytes. PLoS ONE.

[14] Oqani R.K., Lin T., Lee J.E., Kim S.Y., Kang J.W., Jin D.I. (2017). Effects of CDK inhibitors on the maturation, transcription, and MPF activity of porcine oocytes. Reprod. Biol..

[15] Belli M., Shimasaki S. (2018). Molecular aspects and clinical relevance of GDF9 and BMP15 in ovarian function. Vitam. Horm..

[16] Lee S.H., Oh H.J., Kim M.J., Setyawan E.M.N., Lee B.C. (2019). Interaction of the EGFR signaling pathway with porcine cumulus oocyte complexes and oviduct cells in a coculture system. J. Cell. Physiol..

[17] Tamura H., Nakamura Y., Terron M.P., Flores L.J., Manchester L.C., Tan D.X., Sugino N., Reiter R.J. (2008). Melatonin and pregnancy in the human. Reprod. Toxicol..

[18] Yang L., Wang Q., Cui M., Li Q., Mu S., Zhao Z. (2020). Effect of Melatonin on the in vitro maturation of porcine oocytes, development of parthenogenetically activated embryos, and expression of genes related to the oocyte developmental capability. Animals.

[19] Li Y., Zhang Z., He C., Zhu K., Xu Z., Ma T., Tao J., Liu G. (2015). Melatonin protects porcine oocyte in vitro maturation from heat stress. J. Pineal Res..

[20] Cavallari F.C., Leal C.L.V., Zvi R., Hansen P.J. (2019). Effects of melatonin on production of reactive oxygen species and developmental competence of bovine oocytes exposed to heat shock and oxidative stress during in vitro maturation. Zygote.

[21] Shi J.M., Tian X.Z., Zhou G.B., Wang L., Gao C., Zhu S.E., Zeng S.M., Tian J.H., Liu G.S. (2009). Melatonin exists in porcine follicular fluid and improves in vitro maturation and parthenogenetic development of porcine oocytes. J. Pineal Res..

[22] Li Y., Wang J., Zhang Z., Yi J., He C., Wang F., Tian X., Yang M., Song Y., He P. (2016). Resveratrol compares with melatonin in improving in vitro porcine oocyte maturation under heat stress. J. Anim. Sci. Biotechnol..

[23] Livak K.J., Schmittgen T.D. (2001). Analysis of relative gene expression data using real-time quantitative PCR and the 2(-Delta Delta C(T)) method. Methods.

[24] Cao Z., Gao D., Tong X., Xu T., Zhang D., Wang Y., Liu Y., Li Y., Zhang Y., Pu Y. (2019). Melatonin improves developmental competence of oocyte-granulosa cell complexes from porcine preantral follicles. Theriogenology.

[25] Hartig S.M. (2013). Basic image analysis and manipulation in ImageJ. Curr. Protoc. Mol. Biol..

[26] Li R., Albertini D.F. (2013). The road to maturation: Somatic cell interaction and self-organization of the mammalian oocyte. Nat. Rev. Mol. Cell Biol..

[27] Cruz M.H., Leal C.L., da Cruz J.F., Tan D.X., Reiter R.J. (2014). Role of melatonin on production and preservation of gametes and embryos: A brief review. Anim. Reprod. Sci..

[28] Wu T., Lane S.I.R., Morgan S.L., Jones K.T. (2018). Spindle tubulin and MTOC asymmetries may explain meiotic drive in oocytes. Nat. Commun..

[29] Liu H., Yin F.X., Bai C.L., Shen Q.Y., Wei Z.Y., Li X.X., Liang H., Bou S., Li G.P. (2013). TFIIB co-localizes and interacts with α-tubulin during oocyte meiosis in the mouse and depletion of TFIIB causes arrest of subsequent embryo development. PLoS ONE.

[30] Serra E., Succu S., Berlinguer F., Porcu C., Leoni G.G., Naitana S., Gadau S.D. (2018). Tubulin posttranslational modifications in in vitro matured prepubertal and adult ovine oocytes. Theriogenology.

[31] Lee J., Miyano T., Moor R.M. (2000). Spindle formation and dynamics of gamma-tubulin and nuclear mitotic apparatus protein distribution during meiosis in pig and mouse oocytes. Biol. Reprod..

[32] He Y.T., Wang W., Shen W., Sun Q.Y., Yin S. (2019). Melatonin protects against Fenoxaprop-ethyl exposure-induced meiotic defects oocytes. Toxicology.

[33] Ju J.C., Tseng J.K. (2004). Nuclear and cytoskeletal alterations of in vitro matured porcine oocytes under hyperthermia. Mol. Reprod. Dev..

[34] Wang H., Luo J., Carlton C., McGinnis L.K., Kinsey W.H. (2017). Sperm-oocyte contact induces outside-in signaling via PYK2 activation. Dev. Biol..

[35] Yin C., Liu J., He B., Jia L., Gong Y., Guo H., Zhao R. (2019). Heat stress induces distinct responses in porcine cumulus cells and oocytes associated with disrupted gap junction and trans-zonal projection colocalization. J. Cell. Physiol..

[36] Dai X., Lu Y., Zhang M., Miao Y., Zhou C., Cui Z., Xiong B. (2017). Melatonin improves the fertilization ability of post-ovulatory aged mouse oocytes by stabilizing ovastacin and Juno to promote sperm binding and fusion. Hum. Reprod..

[37] Neuer A., Spandorfer S.D., Giraldo P., Dieterle S., Rosenwaks Z., Witkin S.S. (2000). The role of heat shock proteins in reproduction. Hum. Reprod. Update.

[38] Khan I., Lee K.L., Xu L., Mesalam A., Chowdhury M.M., Joo M.D., Ihsan-Ul-Haq, Mirza B., Kong I.K. (2017). Improvement of in vitro-produced bovine embryo treated with coagulansin-A under heat-stressed condition. Reproduction.

[39] Souza-Cácares M.B., Fialho A.L.L., Silva W.A.L., Cardoso C.J.T., Pöhland R., Martins M.I.M., Melo-Sterza F.A. (2019). Oocyte quality and heat shock proteins in oocytes from bovine breeds adapted to the tropics under different conditions of environmental thermal stress. Theriogenology.

[40] Manejwala F.M., Logan C.Y., Schultz R.M. (1991). Regulation of hsp70 mRNA levels during oocyte maturation and zygotic gene activation in the mouse. Dev. Biol..

[41] Pang Y., Zhao S., Sun Y., Jiang X., Hao H., Du W., Zhu H. (2018). Protective effects of melatonin on the in vitro developmental competence of bovine oocytes. Anim. Sci. J..

[42] Tseng J.K., Tang P.C., Ju J.C. (2006). In vitro thermal stress induces apoptosis and reduces development of porcine parthenotes. Theriogenology.

[43] Bellezza I., Giambanco I., Minelli A., Donato R. (2018). Nrf2-Keap1 signaling in oxidative and reductive stress. Biochim. Biophys. Acta Mol. Cell. Res..

[44] Ma R., Liang W., Sun Q., Qiu X., Lin Y., Ge X., Jueraitetibaike K., Xie M., Zhou J., Huang X. (2018). Sirt1/Nrf2 pathway is involved in oocyte aging by regulating Cyclin B1. Aging.

[45] Wang C., Zhou Y.L., Zhu Q.H., Zhou Z.K., Gu W.B., Liu Z.P., Wang L.Z., Shu M.A. (2018). Effects of heat stress on the liver of the Chinese giant salamander Andrias davidianus: Histopathological changes and expression characterization of Nrf2-mediated antioxidant pathway genes. J. Therm. Biol..

[46] Wang Y., Yang C., Elsheikh N.A.H., Li C., Yang F., Wang G., Li L. (2019). HO-1 reduces heat stress-induced apoptosis in bovine granulosa cells by suppressing oxidative stress. Aging.

[47] Kim E.H., Kim G.A., Taweechaipaisankul A., Lee S.H., Qasim M., Ahn C., Lee B.C. (2019). Melatonin enhances porcine embryo development via the Nrf2/ARE signaling pathway. J. Mol. Endocrinol..

[48] Kim E.H., Ridlo M.R., Lee B.C., Kim G.A. (2020). Melatonin-Nrf2 signaling activates peroxisomal activities in porcine cumulus cell-oocyte complexes. Antioxidants.

[49] Li J., Ouyang Y.C., Zhang C.H., Qian W.P., Sun Q.Y. (2019). The cyclin B2/CDK1 complex inhibits separase activity in mouse oocyte meiosis I. Development.

[50] Xu G., Stevens S.M., Kobeissy F., Brown H., McClung S., Gold M.S., Borchelt D.R. (2012). Identification of proteins sensitive to thermal stress in human neuroblastoma and glioma cell lines. PLoS ONE.

[51] Liu L., Xu Y., Reiter R.J. (2013). Melatonin inhibits the proliferation of human osteosarcoma cell line MG-63. Bone.

[52] Sanfins A., Rodrigues P., Albertini D.F. (2018). GDF-9 and BMP-15 direct the follicle symphony. J. Assist. Reprod. Genet..

[53] Riepsamen A.H., Chan K., Lien S., Sweeten P., Donoghoe M.W., Walker G., Fraison E.H.J., Stocker W.A., Walton K.L., Harrison C.A. (2019). Serum Concentrations of Oocyte-Secreted Factors BMP15 and GDF9 During IVF and in Women with Reproductive Pathologies. Endocrinology.

[54] Lee S.H., Oh H.J., Kim M.J., Kim G.A., Choi Y.B., Jo Y.K., Setyawan E.M.N., Lee B.C. (2018). Effect of co-culture canine cumulus and oviduct cells with porcine oocytes during maturation and subsequent embryo development of parthenotes in vitro. Theriogenology.

[55] Gendelman M., Roth Z. (2012). In vivo vs. in vitro models for studying the effects of elevated temperature on the GV-stage oocyte, subsequent developmental competence and gene expression. Anim. Reprod. Sci..

[56] Tian X., Wang F., He C., Zhang L., Tan D., Reiter R.J., Xu J., Ji P., Liu G. (2014). Beneficial effects of melatonin on bovine oocytes maturation: A mechanistic approach. J. Pineal Res..

